# Single-Pixel Shortwave Infrared Imaging Based on PbS Quantum Dots

**DOI:** 10.3390/ma19173719

**Published:** 2026-08-31

**Authors:** Jingbo Li, Guopeng Li, Jiawei Wei, Zhenxiang Gao, Pengfei Xiang, Zhe Wang, Xiaokun Yang, Xudong Mao, Jie Chen, Yong Xia

**Affiliations:** 1 Jiangxi General Institute of Testing and Certification, Nanchang 330052, China; 2 School of Information Engineering, Nanchang University, Nanchang 330031, China; 3 School of Physics and Materials Science, Nanchang University, Nanchang 330031, China; 4 Wuhan National Laboratory for Optoelectronics, Huazhong University of Science and Technology, Wuhan 430074, China

**Keywords:** quantum dot, infrared system, amplification circuit, single pixel imaging

## Abstract

Shortwave infrared (SWIR) imaging technology, with its excellent penetration and anti-interference capabilities, is widely applied in military, medical, and industrial fields. However, traditional detectors (e.g., InGaAs) are expensive, have complex readout circuits, and exhibit insufficient low-light performance, limiting their large-scale promotion. This paper proposes and implements a single-pixel SWIR imaging system based on PbS quantum dot (QD) detectors. A single-pixel imaging system is constructed using PbS QD detectors with a formal device structure (ITO/ZnO/PbS/PbS-EDT/Au); through simulation studies, the effect of the PbS absorption layer thickness on device performance is investigated, and it is determined that a thickness of 450 nm yields optimal device performance. Based on the simulation results, a P-I-N structure PbS photovoltaic-type detector with high external quantum efficiency (*EQE*) and low dark current is fabricated, achieving a *EQE* of 62% at the 1300 nm wavelength, a dark current density of 8.54 × 10^−4^ mA·cm^−2^ at −0.1 V bias voltage, and a −3 dB bandwidth of 324 kHz; a low-noise signal conditioning circuit is designed to optimize the −3 dB bandwidth to 337 kHz while maintaining low noise density, enabling the linear conversion of nA~μA level weak photocurrent from the detector to 0~3 V standardized voltage signals, meeting the requirements of single-pixel imaging (SPI) systems. Hadamard orthogonal encoding technology is employed to achieve spatial light modulation and signal encoding; after the PbS QD detector collects and integrates the projection signal, the image with 128 × 128 resolution is reconstructed through the inverse Hadamard orthogonal decoding algorithm. This work provides a novel solution for QD-based SWIR imaging, overcoming the cost and manufacturing limitations of traditional array systems and laying the foundation for the spectral expansion and practical application of SPI technology. Quantitative imaging characterization and low-light imaging tests are supplemented to verify the comprehensive performance of the system.

## 1. Introduction

Shortwave infrared (SWIR) imaging technology, with its exceptional penetration [1,2], night vision capabilities [3], and stealth, has been widely applied in fields such as military reconnaissance [4], medical diagnosis [5], autonomous driving [6], and agricultural monitoring [7]. Current mainstream imaging solutions are divided into array imaging and scanning imaging. However, both types of imaging are limited by factors such as structural design, manufacturing processes, cost, and application scenarios, creating an opportunity for the rise of single-pixel imaging technology. As a new computational imaging technology, SPI can acquire two-dimensional images of targets by combining a single detector with light field modulation and reconstruction algorithms, addressing the pain points of traditional solutions: First, it discards array structures, eliminating the need for complex integration processes, significantly reducing system costs, and adapting to special bands such as extremely low light and SWIR. Second, it offers excellent temporal resolution, and when combined with fast algorithms, it can shorten imaging time, avoiding the synchronization challenges of scanning imaging, while accommodating both static and dynamic target imaging, making it an ideal solution to break through traditional technological bottlenecks.

Indium Gallium Arsenide (InGaAs) photodetectors (PDs), with their low dark current, high sensitivity, and stability, remain the mainstream technology for SWIR imaging. However, InGaAs chips face challenges in readout integrated circuit (ROIC) integration [8,9], relying on complex epitaxial growth and flip-chip bonding processes, making them expensive in the market, and requiring cooling, which leads to high system power consumption and size, making them difficult to meet the demands of portable, low-power applications. In recent years, advancements in lead sulfide QD technology have provided a promising alternative for SWIR imaging [10,11,12,13,14,15]. Solution-processable PbS QDs enable convenient ROIC integration, achieve tunable spectral response through particle size modulation, and enhance photoelectric performance through surface modification and ligand engineering [16,17,18]. As a result, SWIR detection technology based on PbS QDs has gained significant momentum.

Compared with traditional bulk PbS infrared detectors, colloidal PbS QD thin films exhibit obvious inherent advantages: (1) Tunable bandgap: the spectral response can be precisely matched to the target SWIR band by adjusting the QD diameter, while bulk PbS owns a fixed bandgap without adjustability. (2) Low-cost solution-processable fabrication: QD thin films can be spin-coated at room temperature, whereas bulk single-crystal PbS requires high-temperature epitaxial growth. (3) Superior noise and temperature stability: ligand-optimized QD films possess lower bulk trap density, delivering lower shot noise at room temperature, and their dark current rises far more slowly with temperature than commercial bulk PbS devices.

Current PbS QD imaging research focuses on ROIC-interconnected array systems. Pejovic et al. demonstrated a 768 × 512-resolution sensor using a modified 130 nm CMOS process with integrated colloidal quantum dot (CQD) thin-film photodiodes [19]. Jiang Tang’s group achieved 640 × 512 pixel imaging by optimizing transparent conductive oxide deposition and integrating CQD detectors with CMOS ROICs [20]. Liu et al. further enhanced device performance via planar cation passivation, enabling 64 × 64 thin-film transistor (TFT) imaging [21]. Despite progress, these approaches depend on costly custom ROICs and wafer-level fabrication, limiting scalability. In contrast, point-scanning systems utilize LED/laser illumination and mechanically scanned single-pixel detectors to bypass ROIC requirements, reducing costs. Hwang et al. demonstrated “KIST” letter imaging using a PbS QD/IGZO hybrid detector on an X-Z stage [22], while Hyun Tae Choi’s team achieved “duck” imaging via a 1 × 6 line scanner [23]. Ge Mu et al. extended this to room-temperature mid-wave infrared imaging using barrier heterojunction detectors [24]. However, line-by-line pixel scanning inherently suffers from slow speed and bulky instrumentation, making it unsuitable for high-resolution applications, driving demand for economical and compact alternatives.

In this context, SPI systems, with their unique imaging mechanism, have become a highly promising alternative solution to address the aforementioned technical bottlenecks. Unlike array systems that rely on ROIC, SPI systems do not require the fabrication of large-scale pixel arrays; instead, they achieve complete two-dimensional or even three-dimensional imaging through a single or a few detectors, combined with structured illumination and computational reconstruction algorithms, fundamentally avoiding the high costs and low scalability challenges of custom ROIC and wafer-level manufacturing. At the same time, compared to traditional point-scan systems, SPI systems do not require complex mechanical scanning structures, effectively overcoming limitations such as slow speed and bulky instruments, enabling the miniaturization, lightweighting, and high-speed operation of the system while maintaining imaging quality. Zhang et al. reported a 70% cost reduction in UV cameras using SPI [25], while Xianlin Song et al. enabled rapid high-resolution reconstruction via diffusion modeling [26]. Sun Baoqing’s team combined PbS filters with InGaAs detectors for hyperspectral SPI [27]. Though SPI reduces costs and imaging time [28,29], most SWIR-SPI systems still employ InGaAs detectors [30], with limited exploration of PbS QDs. A comprehensive benchmark comparison between our system and previously reported quantum-dot-based single-pixel imaging systems is provided in Table 1.

Different from previous quantum-dot single-pixel imaging studies, the core innovations of this work are summarized as follows: First, we construct an integrated system of PIN-structured PbS QD photovoltaic detector and customized wideband low-noise TIA secondary amplification filter circuit, which realizes linear conversion of nA–μA weak photocurrent to 0–3 V standard voltage signals and perfectly matches the hardware requirement of Hadamard single-pixel imaging; second, SCAPS simulation is systematically carried out to optimize the PbS absorber thickness to 450 nm for maximizing SWIR photoelectric conversion efficiency, which is rarely reported in existing QD SPI studies; third, the whole system only adopts solution-processed PbS QD photodiode as the photosensitive unit without expensive InGaAs chips and cooling equipment, significantly reducing the overall system cost.

Combining the current research status of PbS SPI, it still faces core issues such as device performance, system compatibility, and cost control in SWIR applications, which severely restrict scene expansion. To address this, we propose a SWIR-SPI system based on PbS QDs. Key challenges include optimizing the performance of PbS QD detectors and resolving the signal mismatch between detector output and SPI input. PbS QD detectors are selected to build a single-device imaging system. Through software simulation, the influence of PbS absorption layer thickness on device performance is studied to determine the optimal thickness. PbS photovoltaic-type detectors with a P-I-N structure are fabricated, achieving an *EQE* of 62% at 1300 nm and a dark current density of 8.54 × 10^−4^ mA·cm^−2^ at −0.1 V bias, with a −3 dB bandwidth of 324 kHz. A low-noise signal conditioning circuit is designed and built to amplify the weak electrical signals from the detector to a standardized voltage, optimizing the −3 dB bandwidth to 337 kHz. The standardized signal is then fed into the Hadamard SPI system to obtain an image with 128 × 128 resolution. In this work, we provide a low-cost SWIR imaging solution based on single-pixel detectors.

## 2. Detector and Its Performance

### 2.1. Detector Device Structure and Simulation

**Figure 1:**
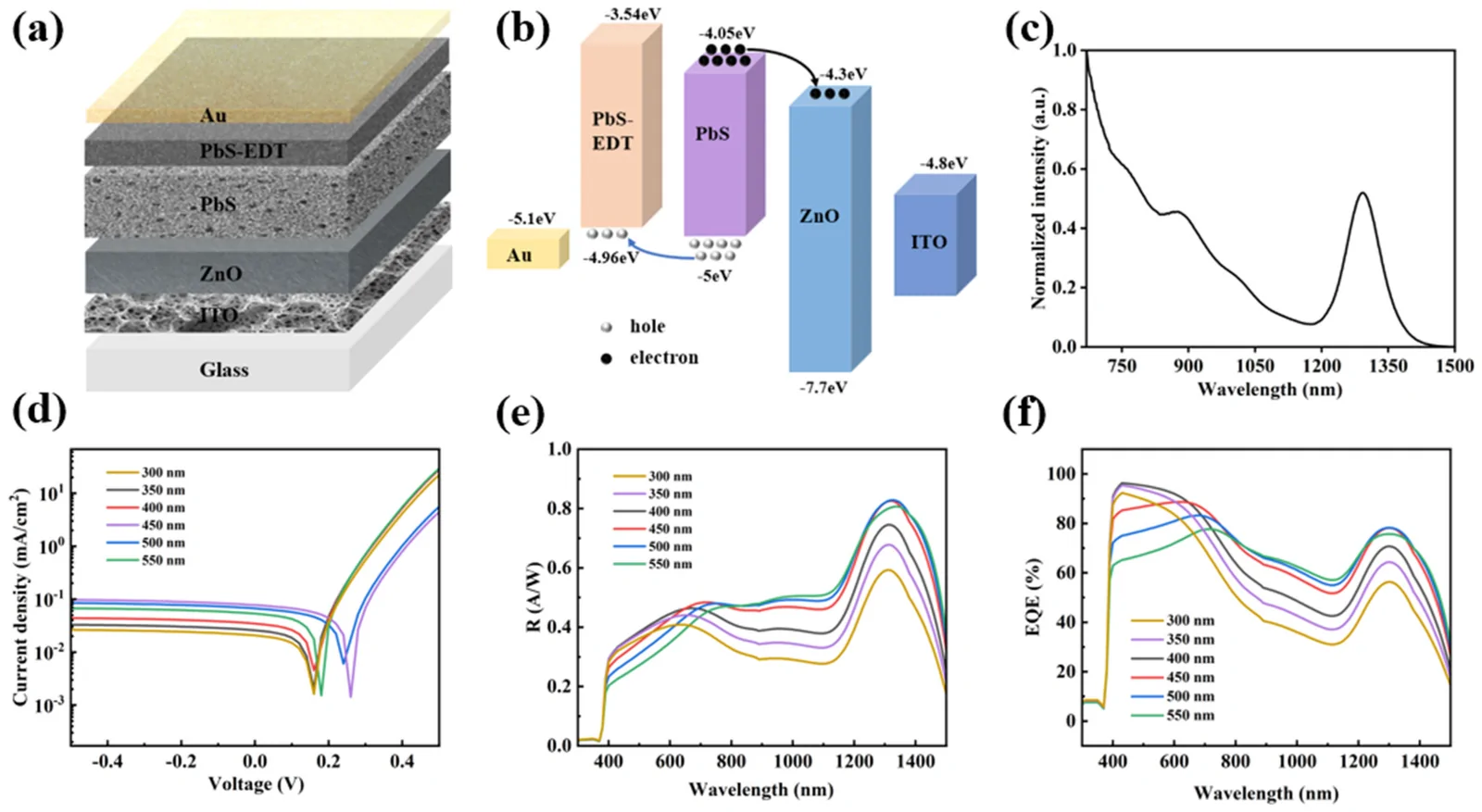
(**a**) Detector device structure. (**b**) Device band diagram. (**c**) Absorption spectrum of PbS. (**d**) *I*–*V* curves for PbS absorber layers of different thicknesses. (**e**) *R* curves for PbS absorber layers of different thicknesses. (**f**) *EQE* curves for PbS absorber layers of different thicknesses. All detailed SCAPS simulation input parameters are summarized in Table S1 of the Supporting Information.

Throughout the entire system, the magnitude of dark current and the response rate of the detector are key factors determining imaging quality and speed. This study employs a specific Au/PbS-EDT/PbS/ZnO/ITO structure to fabricate a P-I-N device configuration, as illustrated in Figure 1a. When the functional layers of the PbS QD detector are not in contact, the theoretical energy levels of the conduction band and valence band are depicted in Figure 1b. As the core functional layer, the thickness parameter of the PbS absorption layer directly impacts the device’s overall performance. Therefore, its optimal thickness must be determined prior to fabrication.

Figure 1c shows the absorption spectrum of the PbS layer. Its first exciton absorption peak is located at 1300 nm, indicating that the device can effectively absorb SWIR light. Therefore, by simulating it using SCAPS simulation software (version 3.3.07) under conditions of 1300 nm wavelength and 1 W·cm^−2^ light intensity, the electrical performance curves of the device under different absorption layer thicknesses were obtained. As depicted in Figure 1d, in which the *I*–*V* curves for different PbS absorber layer thicknesses are shown, increasing the absorber layer thickness from 300 nm to 550 nm causes the current density at a −5 V bias to gradually rise from 0.03 mA·cm^−2^ to a peak of 0.1 mA·cm^−2^, before declining back to 0.05 mA·cm^−2^. As depicted in Figure 1e,f, the responsivity (*R*) curves exhibit consistent trends with the *EQE* curves. Test data confirming these variations with absorption layer thickness further validate that optimal performance occurs at an absorption layer thickness of 450 nm. At this thickness, the device achieves an *R* of 0.82 A·W^−1^ and an *EQE* of 78.2%, maintaining the highest values across the entire wavelength range. The simulation results indicate that a 450 nm PbS absorber layer thickness is optimal for this device structure.

There is an obvious deviation between the simulated EQE (78.2% at 450 nm) and the experimental EQE (62% at 1300 nm), which originates from multiple non-ideal factors in actual device fabrication: (1) Microscopic inhomogeneity and slight local agglomeration of spin-coated PbS QD films introduce a large number of carrier recombination centers; (2) incomplete ligand exchange at the PbS/PbS-EDT interface increases interface carrier recombination loss; (3) optical reflection loss at each functional layer interface is ignored in ideal SCAPS simulation; (4) minor contact resistance loss exists between the Au electrode and PbS-EDT layer.

### 2.2. Component Performance Analysis

**Figure 2:**
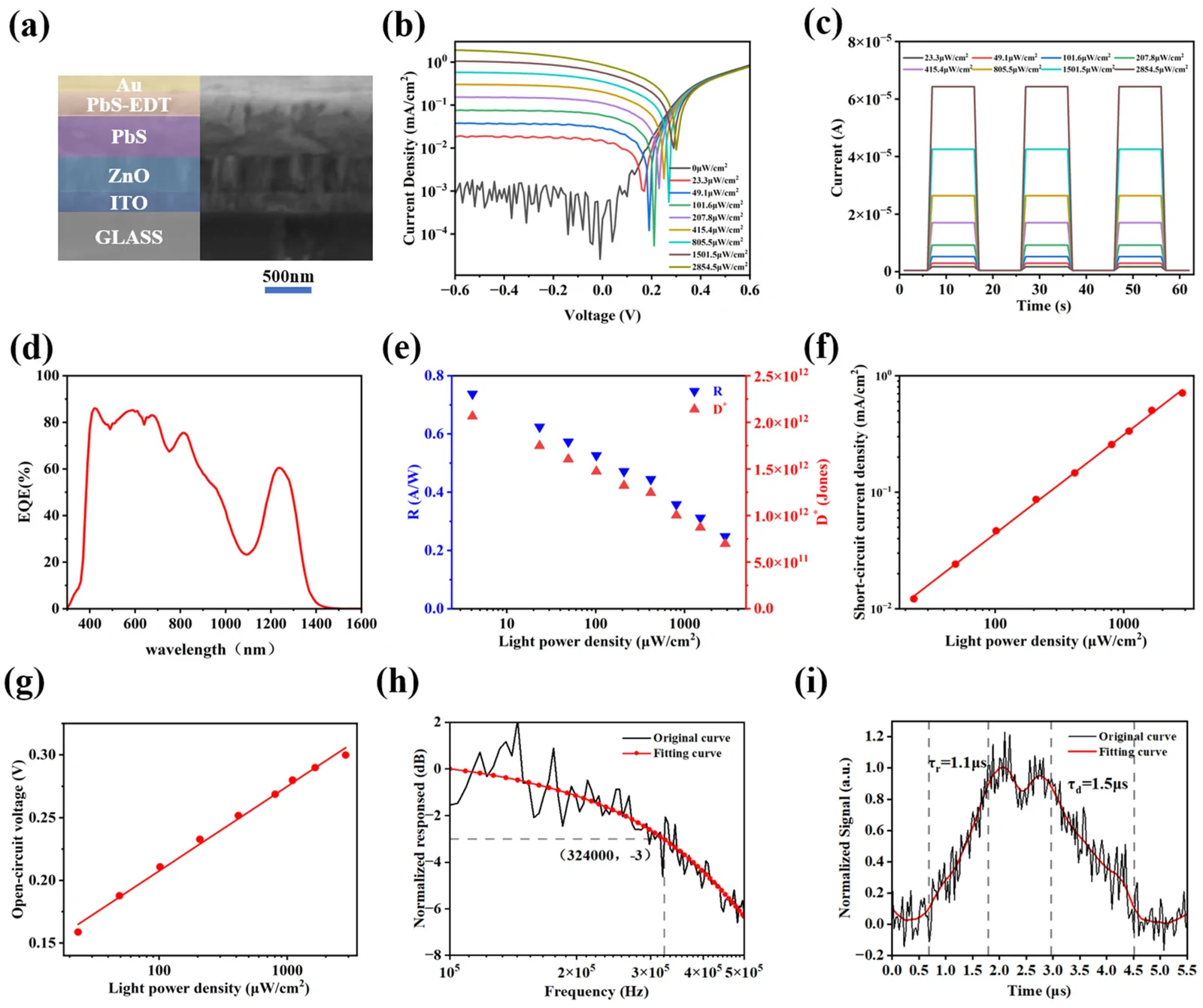
(**a**) SEM image of the PbS PD. (**b**) *I*–*V* curves at 1200 nm wavelength under different optical power densities. (**c**) *I*–*V* curves at 1200 nm wavelength under different optical power densities. (**d**) *EQE* curve of the device. (**e**) *R* and D* at 1200 nm under different optical power densities. The D* value here is a shot-noise-limited theoretical value. (**f**) Short-circuit current density versus optical power density. (**g**) Open-circuit voltage versus optical power density. (**h**) Frequency response curve of the detector. (**i**) Rise and decay times.

Based on the aforementioned simulations, we fabricated a PbS QD detector with a 450 nm thick PbS absorption layer. Figure 2a shows the SEM image of the detector, and performance testing was conducted on the entire device. Figure 2b displays the *I*–*V* characteristic curves of the detector under different light intensities at a wavelength of 1200 nm. Under dark conditions, the dark current density at a −0.1 V bias was 8.54 × 10^−4^ mA·cm^−2^. The photocurrent increased linearly with light intensity, reaching a current density of 0.13 mA·cm^−2^ when the incident light intensity reached 207.8 μW·cm^−2^. As shown in Figure 2c, the *I*–*T* curves at different optical power densities indicate that the current increases to 1.7 × 10^−6^ A at an illumination intensity of 23.3 μW·cm^−2^, while the dark current remains at an extremely low level throughout the measurement process. External *EQE*, a key performance metric for photodetectors, is defined as the ratio of collected electrons to incident photons. As shown in Figure 2d, *EQE* reached 62% at 1300 nm wavelength, indicating the device’s high efficiency in converting SWIR light into electrical signals. This demonstrates the device’s significant response characteristics and excellent repeatability across varying light intensities.

Under a bias voltage of −0.1 V, the device’s *R* and specific detectivity (D*) are shown in Figure 2e. *R*, a key parameter characterizing the sensitivity of photodetectors, is defined as Equation (1):

(1) $$R=\frac{I_{ph}}{P\times S}$$

where *I_ph_* represents the photocurrent generated by illumination, *P* denotes the incident light power density, and *S* corresponds to the effective area of the photodetector. D* characterizes the ability to detect weak light and can be calculated using Equation (2):

(2) $$D^{*}=\frac{R}{(2q\times I_{dark}{)}^{\frac{1}{2}}}$$

where *I_dark_* is the detector’s dark current. The calculated D* of 2.06 × 10^12^ Jones at 4.1 μW·cm^−2^ is a shot-noise-limited theoretical value, only considering dark current shot noise under −0.1 V bias. All calculation assumptions are clarified as follows: (1) Shot noise dominates within the effective imaging bandwidth of 50–337 kHz; (2) no stray light interference exists during testing; (3) the effective photosensitive area is precisely calibrated by an optical power meter; (4) all tests are performed at room temperature (25 °C) without cooling. Results show that at an incident light power density of 4.1 μW·cm^−2^, the relative detection rate reaches 2.06 × 10^12^ Jones, corresponding to a responsivity of 0.73 A·W^−1^.

As a PIN-structured photovoltaic detector, the short-circuit current density and open-circuit voltage reflect its “self-powering capability.” Higher values indicate superior performance without external bias. As shown in Figure 2f,g, both short-circuit current density and open-circuit voltage exhibit a positive proportional relationship with light power density. At an incident optical power density of 23.3 μW·cm^−2^, the short-circuit current density reached 0.012 mA·cm^−2^ and the open-circuit voltage reached 0.16 V, both exhibiting favorable response characteristics.

Figure 2h displays the device’s −3 dB bandwidth characteristic, showing signal attenuation to −3 dB at a frequency of 324 kHz. Figure 2i presents the rise time and fall time of the PbS photodiode (defined as the transition time from 10% to 90% signal amplitude), with measured values of 1.1 μs and 1.5 μs, respectively. The bandwidth frequency calculated using Equation (3):

(3) $$f=\frac{0.35}{\tau_{r}}$$

is 318 kHz, which falls within an acceptable error range.

## 3. Circuit Analysis and Design

**Figure 3:**
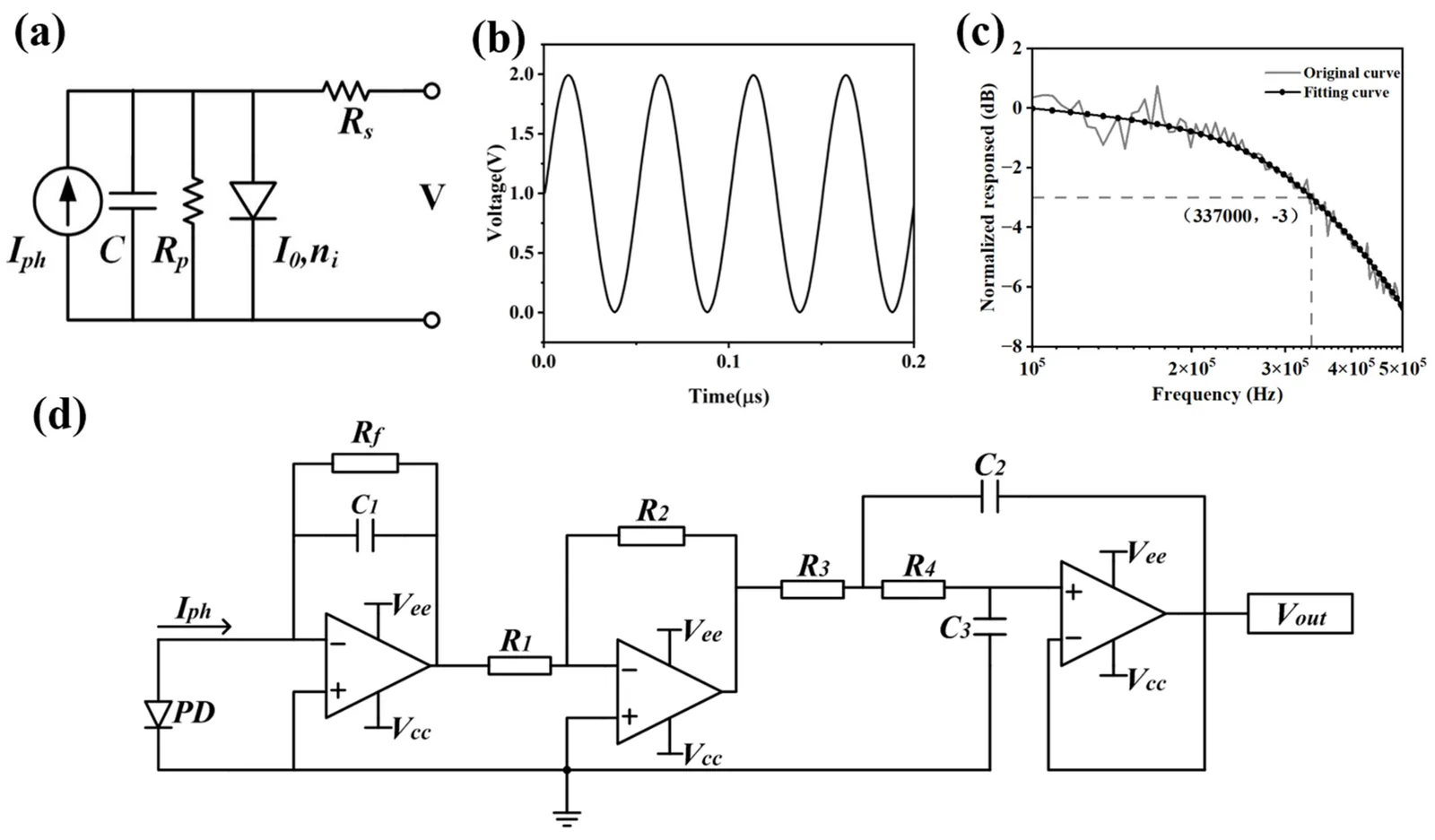
(**a**) Equivalent circuit model of the device. (**b**) Amplification curve for a 1 μA sinusoidal signal. (**c**) System frequency response curve. (**d**) Schematic of the matching circuit. All circuit component parameters and op-amp models are summarized in Table S2 of Supporting Information.

The above data confirms the outstanding performance of this detector. However, since its output is a weak current signal incompatible with the voltage signal required for SPI, a dedicated circuit must be designed to achieve signal matching. The equivalent circuit model of the device is crucial for selecting an appropriate circuit configuration. This study employs mainstream circuit models for analysis [31], as shown in Figure 3a. Typically, *R_p_* is much larger than *R_s_*.

Current-to-voltage conversion circuits primarily employ two topologies. Resistive Detection Topology: A sampling resistor (*R*) is directly connected to the detector output, converting the current signal into a proportional voltage signal for subsequent amplification. Transimpedance amplifier (TIA) topology: A TIA structure employing an operational amplifier. The TIA utilizes the virtual short and virtual open characteristics of operational amplifiers. The inverting input node forms virtual ground, leading to an extremely low effective input impedance close to 0 Ω. This feature minimizes shunt current loss on the photodiode parallel resistance *R_p_* and avoids signal attenuation caused by load division, fully retaining the intrinsic photoelectric response of PbS QD detectors.

Considering the weak output signal from the detector, the first circuit approach requires a larger series resistor *R*. This would cause the photodiode’s output signal to be divided by the resistor *R_p_*. This study employs a TIA topology as the current-to-voltage conversion circuit, with the overall circuit principle shown in Figure 3d. Within the entire receiver system, the TIA serves as the core current-to-voltage conversion module, centered around an operational amplifier and paired with the feedback resistor *R_f_*. Leveraging the operational amplifier’s “virtual short” and “virtual open” characteristics, it directs nearly all of the nA- to μA-level photocurrent *I_ph_* generated by light excitation in the PbS detector into the feedback resistor. This produces a voltage signal that is linearly proportional to the input current, which satisfies Equation (4):

(4) $$V_{out}=-I_{ph}\times R_{f}$$

where *R_f_* represents the transimpedance gain.

To reduce noise and enhance detection sensitivity, the circuit often requires sacrificing some gain, which can adversely affect subsequent signal processing. Therefore, the signal output from the circuit requires secondary amplification. This solution employs a negative feedback amplifier circuit as the secondary amplification stage, specifically connecting the output signal of the transimpedance amplifier to the inverting input of the main amplifier circuit. The gain of this main amplifier circuit can be calculated using Equation (5):

(5) $$A_{u}=-\frac{R_{2}}{R_{1}}R_{f}$$

The total cascade output voltage of the two-stage amplifier system is revised as Equation (6):

(6) $$V_{out}=I_{ph}\times R_{f}\times \frac{R_{2}}{R_{1}}$$

The total transimpedance gain of the whole circuit is *R_f_* · *R*_2_/*R*_1_ = 4 × 10^5^ V/A, which converts 100 nA photocurrent into a 40 mV linear voltage signal, with the maximum output swing limited to 0–3 V to perfectly match the DAQ input range. The circuit configuration not only effectively amplifies the converted voltage signal but also performs signal inversion. Ultimately, it achieves a significant gain enhancement across the entire signal chain. As shown in Figure 3b, a 1 μA sinusoidal signal undergoes conversion and amplification through this circuit to produce a stable voltage sinusoidal signal. Additionally, to suppress noise interference introduced by ambient light and electronic components, a second-order Butterworth low-pass filter is added downstream of the secondary amplification circuit.

To meet the stringent signal quality requirements of SPI systems, this circuit prioritizes low noise, wide bandwidth, and high linearity in its design. It employs a low-noise operational amplifier and optimizes the precise resistance value of feedback resistor R_f_ to maintain stable, low circuit noise density. Compensation capacitors are introduced into the feedback loop to suppress high-frequency oscillations and counteract parasitic capacitance effects. This enhances the circuit’s −3 dB bandwidth to 337 kHz, as shown in Figure 3c, exceeding the 324 kHz bandwidth of the PbS QD detector. This ensures the detector’s high-frequency response characteristics are transmitted without attenuation. Ultimately, this circuit achieves linear conversion of the detector’s nA~μA level weak photocurrent into standardized 0~3 V voltage signals. This not only perfectly matches the input signal amplitude requirements of SPI systems but also maximally preserves the excellent original signal characteristics of the detector, such as its high EQE and low dark current, laying a high-quality signal foundation for Hadamard-encoded integration projection signal acquisition and high-resolution SWIR image reconstruction. 

There exists a prominent trade-off among circuit gain, bandwidth, and noise. Increasing feedback resistance *R_f_* improves transimpedance gain, but increases the parasitic RC time constant to reduce circuit bandwidth, and simultaneously raises the thermal noise of the feedback resistor. We select *R_f_* = 40 kΩ after comparative tests to balance the three indicators, finally achieving a 337 kHz bandwidth.

## 4. System Testing

We will conduct verification experiments by integrating detectors with circuitry. An LED light source serves as the emitter, while a PbS QD detector functions as the receiver, subsequently connected to a circuit for signal conversion and amplification. We employed a signal generator to control the LED light source’s brightness and emission frequency, simulating relevant scenarios. Through practical testing, we set up DC signals and sinusoidal signals of different frequencies to control the LED light source. Using an oscilloscope to detect the system’s output voltage signal, results showed that when the entire system effectively received SWIR light and the input light remained stable, the system could efficiently output amplified voltage signals. When adjusting the incident light frequency, the system still amplified the effective signal from the incident light.

**Figure 4:**
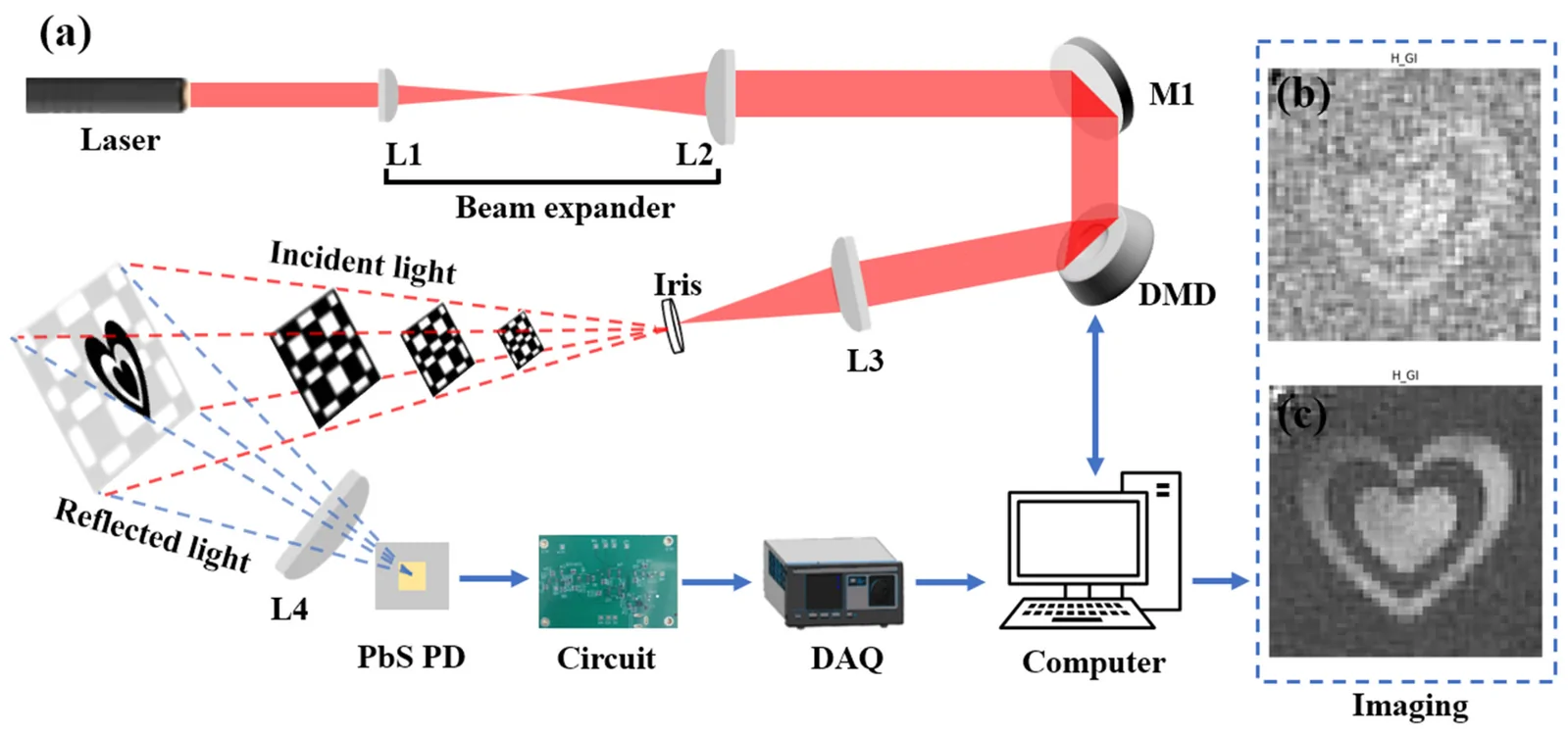
(**a**) Schematic of SPI using a PbS quantum dot detector. (**b**) Image obtained without a matching circuit. (**c**) Image obtained with a matching circuit. Imaging performance under gradient optical power and transmission-mode imaging results are provided in Figures S4 and S6 of the Supporting Information. The total acquisition time for a single sampled 128 × 128 image is 40 s.

We integrated the PbS QD detector with a matching circuit into an SPI system to demonstrate its practical application potential. Using a laser as the light source, the light was directed onto the DMD after lens adjustments. Preprocessed Hadamard-encoded patterns were loaded onto the DMD via computer, then projected onto an object. The reflected light from the object was received by the PbS QD detector. The detector signal is amplified and processed by the matching circuit before being input to a data acquisition (DAQ) system. The acquired data undergoes inverse Hadamard orthogonal decoding on a PC to reconstruct a 128 × 128 resolution image. Figure 4a illustrates the schematic of the entire imaging system. Figure 4b shows the result without the matching circuit, while Figure 4c shows the result with the matching circuit enabled. Comparison reveals that Figure 4c exhibits sharper pixels and significantly reduced noise.

Quantitative image quality metrics (SNR, PSNR, SSIM, Average Contrast) in Table 2 quantitatively prove that the designed low-noise conditioning circuit significantly suppresses system noise and improves image fidelity. The total acquisition time for a single 128 × 128 fully Hadamard sampled image is 40 s, including DMD pattern projection, signal integration, DAQ sampling, and data transmission. The integration time for a single pattern is 25 ms, accounting for 10% of the full sampling, with a total of 1600 patterns being acquired and integrated. Imaging performance under different incident optical powers is presented in Figure S4. The minimum detectable optical power for distinguishable target reconstruction is 0.1 mW, fully demonstrating the excellent low-light imaging capability of the proposed system.

To further characterize the spatial resolution of the system, transmission-mode single-pixel imaging experiments are supplemented, as shown in Figure S6 of Supporting Information. The SWIR laser passes through a resolution transmission mask, and the transmitted light is received by a PbS photodetector for Hadamard reconstruction. The line width mask test shows the minimum distinguishable line width of the system is 0.7931 p/mm, which intuitively reflects the spatial resolution performance. Both reflection and transmission imaging geometries confirm the excellent comprehensive imaging capability of our PbS QD SPI system. This result confirms the broad application potential of PbS QD photodetectors in low-cost single-pixel SWIR imaging systems.

## 5. Conclusions

In this work, we first applied PbS QD photodiodes to a Hadamard SPI system and successfully developed a low-cost SWIR imaging system. A PbS QD detector with the formal device structure (ITO/ZnO/PbS/PbS-EDT/Au) was selected to build the unit device imaging system. SCAPS software simulation determined that the device performed optimally with a PbS absorption layer thickness of 450 nm, leading to the fabrication of a PbS detector with a PIN structure. The device demonstrated outstanding performance: a *EQE* of 62% at 1300 nm, a dark current density of 8.54 × 10^−4^ mA·cm^−2^ at −0.1 V bias, and a −3 dB bandwidth of 324 kHz. We specifically designed and constructed a low-noise signal conditioning circuit to amplify the detector’s weak nA~μA level electrical signals to a standardized voltage range of 0~3 V while maintaining low circuit noise density. The −3 dB bandwidth was further optimized to 337 kHz, enabling efficient adaptation between the detector and imaging system. The system spatially modulates SWIR light using a spatial light modulator loaded with Hadamard-encoded patterns. The prepared PbS QD detector collects the integral projection signals corresponding to each encoded pattern. These signals are then reconstructed into a two-dimensional target image via an inverse Hadamard orthogonal decoding algorithm, yielding a 128 × 128 resolution image. This development points to a new direction for low-cost SWIR imaging systems, paving the way for future research on single-pixel spectral imaging based on PbS detectors. Supplementary quantitative imaging characterization, transmission-mode imaging, and systematic low-light imaging tests fully verify the practicability of the proposed PbS QD single-pixel SWIR imaging system. Comprehensive benchmark comparison with previous studies further highlights the advantages of our integrated device-circuit design in cost, response speed, and imaging quality.

## Figures and Tables

**Table 1:** Benchmark comparison with reported QD-based single-pixel imaging systems.

Reference	Detector Core Material	Device Structure	Working Band	Max Resolution	D* (Jones)	System Bandwidth	Sampling Mode	Single Image Acquisition Time	System Cost Level
Ref. [27]	PbS filter + InGaAs	Commercial PD	Vis–SWIR	64 × 64	—	<100 kHz	Fourier	>5 s	High
Ref. [22]	PbS QD /IGZO hybrid	TFT hybrid device	NIR	32 × 32	~10^11^	<50 kHz	Mechanical scanning	>20 s	Medium
This work	Pure PbS QD	PIN photovoltaic diode	1000–1500 nm SWIR	128 × 128	2.06 × 10^12^	337 kHz	Hadamard encoding	40 s	Low

**Table 2:** Quantitative image quality comparison of reconstructed images with and without signal conditioning circuit.

Index	Without Matching Circuit (Figure 4b)	with Matching Circuit (Figure 4c)
SNR (dB)	6.83	19.76
PSNR (dB)	15.21	28.45
SSIM	0.412	0.897
Average Contrast	0.104	0.369

## Data Availability

The original contributions presented in this study are included in the article/Supplementary Materials. Further inquiries can be directed to the corresponding authors.

## Supplementary Materials

The following supporting information can be downloaded at: https://www.mdpi.com/article/10.3390/ma19173719/s1. Figure S1. (a) Short-circuit current density and open-circuit voltage curves for PbS absorber layers at different thicknesses. (b) TEM pattern of PbS QD detector with excellent monodispersity and negligible particle agglomeration. Full SCAPS simulation input parameters are summarized in Table S1. Figure S2. (a) PCB physical specimen. (b) Actual circuit testing scenario. (c) DC signal amplification effect. (d) Amplification effect of sine signals at different frequencies. The designed signal conditioning circuit achieves a −3 dB bandwidth of 337 kHz, which exceeds the 324 kHz bandwidth of the PbS detector to avoid high-frequency signal attenuation. Complete circuit component parameters are listed in Table S2. Figure S3. Shows imaging results under different light source powers at 550 nm green light. (a) Imaging results at 0.1 mW light intensity. (b) Imaging results at 0.7 mW light intensity. Figure S4. Cardioid reconstruction images under gradient shortwave infrared optical power ((a) 0.1 mW, (b) 0.3 mW, (c) 0.5 mW, (d) 0.7 mW), accompanied by a quantitative statistical data table of SNR/SSIM (Table S3); the imaging image at 0.7 mW is used as the reference image for true values. Figure S5. Spectral density of dark noise current of lead sulfide PIN detector, with test frequency range of 0.01 Hz–10 Hz. Figure S6. Complete set of experimental results for transmissive single-pixel imaging: (a) Actual scene image of the transmissive optical path. (b) Transmissive resolution board. (c) Reconstructed image of the line width of the resolution board. Table S1. Full SCAPS simulation input parameters of each functional layer. Table S2. Complete parameter list of the low-noise signal conditioning circuit. Table S3. Quantitative image-quality metrics of reconstructed SWIR images under gradient incident optical power corresponding to Figure S4. The reconstructed image obtained at 0.7 mW is taken as the ground-truth reference. All metrics are calculated pixel-by-pixel from raw 128 × 128 reconstructed gray-scale matrices without manual denoising or brightness adjustment.
