# Supplementary material for: Single-Pixel Shortwave Infrared Imaging Based on PbS Quantum Dots
Source: Materials (Basel). 2026 Aug 31;19(17):3719. doi: 10.3390/ma19173719 (PMC13567258; doi:10.3390/ma19173719)
Supplement: Supplementary file 1 [file materials-19-03719-s001.zip › materials-4501129-supplementary.pdf]

Supporting Information

Single-Pixel Shortwave Infrared Imaging Based on PbS Quantum Dots

Jingbo Li [1,†], Guopeng Li [2,†], Jiawei Wei [3], Zhenxiang Gao [3], Pengfei Xiang [2], Zhe Wang [2], Xiaokun Yang [4], Xudong Mao [1,*], Jie Chen [1,*] and Yong Xia [2,*]

1 Jiangxi General Institute of Testing and Certification, Nanchang 330052, China
2 School of Information Engineering, Nanchang University, Nanchang 330031, China
3 School of Physics and Materials Science, Nanchang University, Nanchang 330031, China
4 Wuhan National Laboratory for Optoelectronics, Huazhong University of Science and Technology, Wuhan 430074, China
* Correspondence: xudong_mao@126.com (X.M.); ncuchenjie@126.com (J.C.); xiayong@ncu.edu.cn (Y.X.)
† These authors contributed equally to this work and should be considered co-first authors.

1. Original Figures with Revised Captions

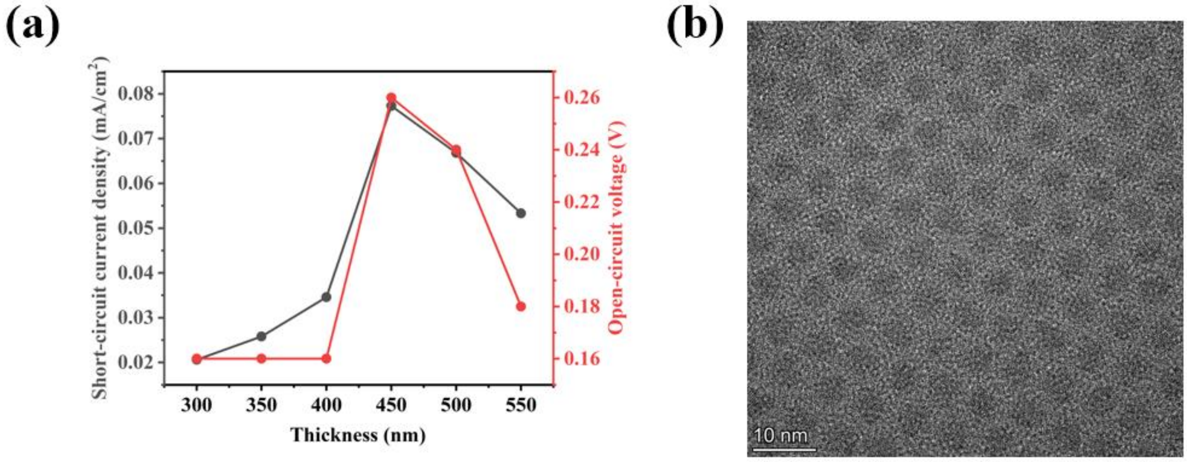

Figure S1. (a) Short-circuit current density and open-circuit voltage curves for PbS absorber layers at different thicknesses. (b) TEM pattern of PbS QD detector with excellent monodispersity and negligible particle agglomeration. Full SCAPS simulation input parameters are summarized in Table S1.

Figure S1a shows the short-circuit current density and open-circuit voltage curves for different thicknesses. As the absorption layer thickness increases from 300 nm to 550 nm, both the short-circuit current density and open-circuit voltage exhibit a trend of first increasing and then decreasing, reaching their maximum values at a thickness of 450 nm. with a short-circuit current density of 0.08 mA/cm^2 and an open-circuit voltage of 0.26 V. When the PbS absorber layer thickness exceeds 450 nm, the short-circuit current density decreases, and the open-circuit voltage follows a similar trend. This relatively confirms that 450 nm is the optimal thickness for the PbS absorber layer. To investigate the particle size distribution and microstructure of PbS quantum dots in depth, transmission electron microscopy (TEM) was employed for detailed characterization. Figure S1b displays TEM images of PbS QDs, revealing excellent monodispersity with no significant agglomeration observed.

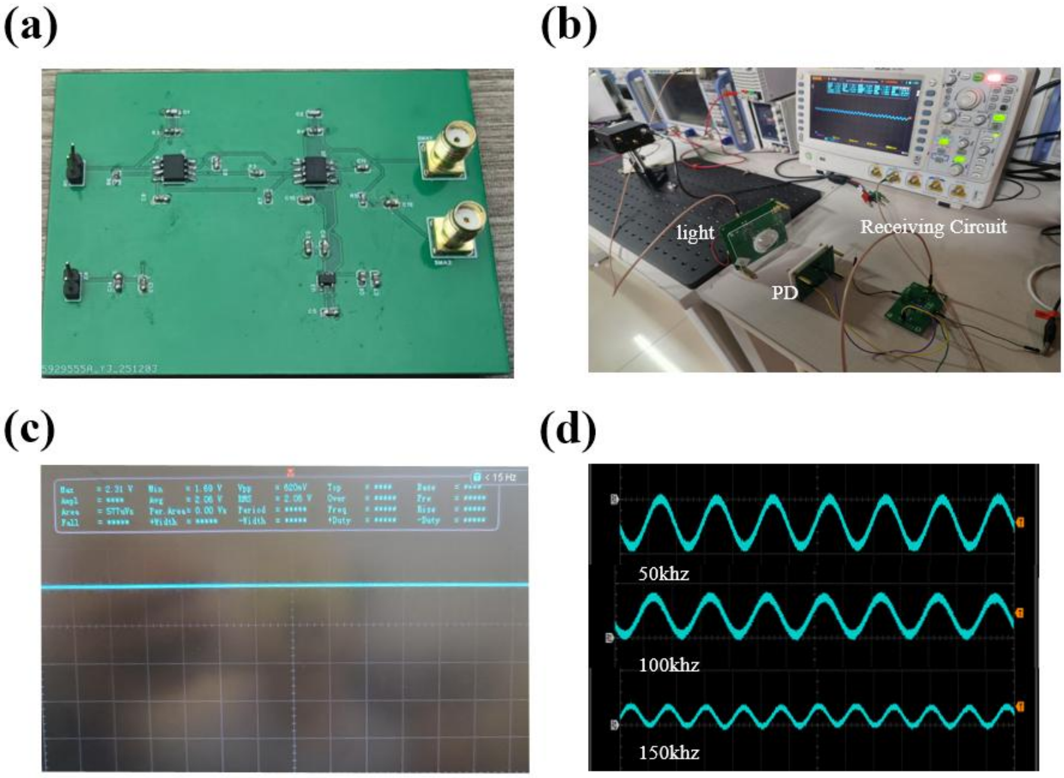

Figure S2. (a) PCB physical specimen. (b) Actual circuit testing scenario. (c) DC signal amplification effect. (d) Amplification effect of sine signals at different frequencies. The designed signal conditioning circuit achieves a -3 dB bandwidth of 337 kHz, which exceeds the 324 kHz bandwidth of the PbS detector to avoid high-frequency signal attenuation. Complete circuit component parameters are listed in Table S2.

We will conduct experimental testing by integrating the detector with the circuit. We use an LED light source as the emitter and a PbS QD detector as the receiver, then connect them to the circuit for signal conversion and amplification. Figure S2a shows the actual test setup. Figure S2b depicts the actual circuit. Observation leads to the conclusion that when the entire system effectively receives SWIR light and the input light remains stable, the system can efficiently output amplified voltage signals, as shown in Figure S2c. When adjusting the incident light frequency, the system still effectively amplifies the signal, as demonstrated in Figure S2d.

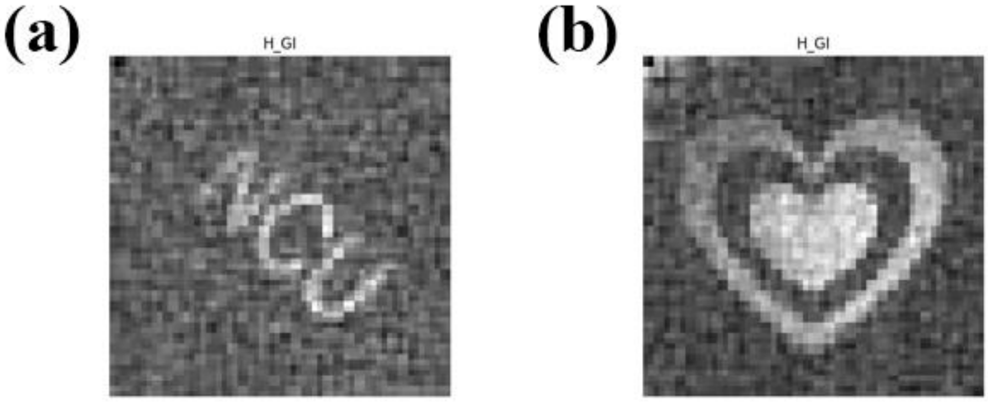

Figure S3. Shows imaging results under different light source powers at 550 nm green light. (a) Imaging results at 0.1 mW light intensity. (b) Imaging results at 0.7 mW light intensity.

The two renderings in Figure S3a and b demonstrate that under different light intensities in the visible spectrum, the system's single-pixel imaging performance yields satisfactory results. This indicates that the system is also capable of performing single-pixel spectral imaging to a certain extent.

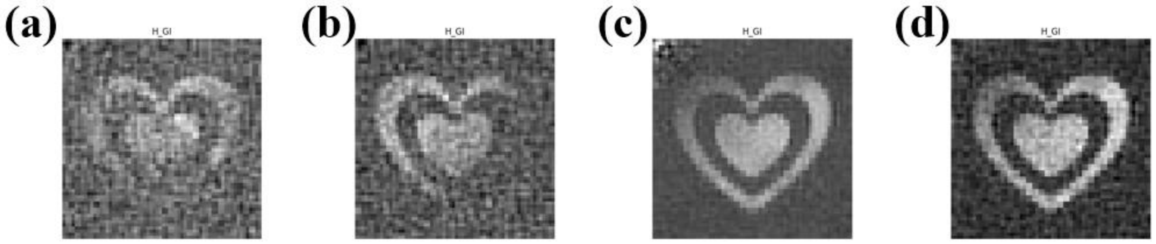

Figure S4. Cardioid reconstruction images under gradient shortwave infrared optical power ((a) 0.1 mW, (b) 0.3 mW, (c) 0.5 mW, (d) 0.7 mW), accompanied by a quantitative statistical data table of SNR/SSIM (Table S3); the imaging image at 0.7 mW is used as the reference image for true values.

Figure S4 systematically characterizes the low-light imaging performance of the PbS QD single-pixel system under short-wave infrared illumination. Four groups of identical heart-shaped targets are reconstructed with only incident SWIR power changed, and all other optical and acquisition parameters remain consistent. Quantitative metrics SNR, PSNR, and SSIM are calculated by taking the highest-brightness 0.7 mW image as the reference standard, and the statistical data are sorted into the attached table below the figure. The result indicates that a distinguishable target contour can be reconstructed at the minimum power of 0.1 mW, quantitatively supporting the low-light imaging advantage proposed in the main text.

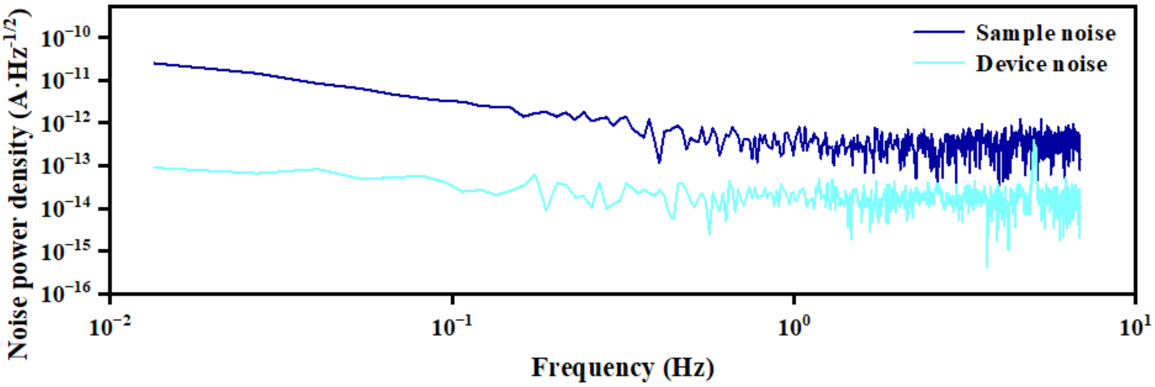

Figure S5. Spectral density of dark noise current of lead sulfide PIN detector, with test frequency range of 0.01 Hz–10 Hz.

Figure S5 shows the dark noise spectrum of the device measured by a semiconductor parameter analyzer; the plot only retains the positive frequency range and employs a double logarithmic coordinate. Due to instrument limitations, the curve clearly indicates that below 10 Hz, 1/f flicker noise is dominant; within the effective imaging bandwidth of the system, which is 50–337 kHz, shot noise is the main noise source.

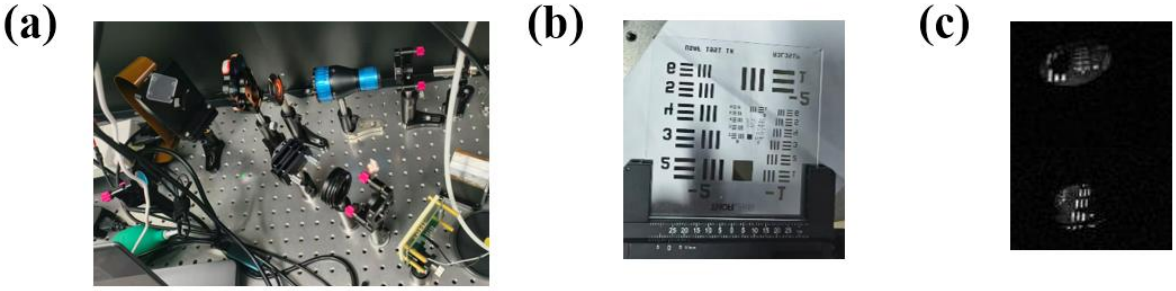

Figure S6. Complete set of experimental results for transmissive single-pixel imaging: (a) Actual scene image of the transmissive optical path. (b) Transmissive resolution board. (c) Reconstructed image of the line width of the resolution board.

Figure S6 Supplementary Transmission Imaging Experiment, as a complement to the reflection imaging in the main text, evaluates the spatial resolution of the system. A short-wave infrared laser passes through the transmission mask and is received and reconstructed by the detector; the minimum resolvable line width measured in the line width mask experiment is 0.7931 p/mm, fully demonstrating the imaging resolution capability of the entire device-circuit integration system.

Table S1. Full SCAPS simulation input parameters of each functional layer.

Parameters	PbS-EDT	PbS	ZnO	ITO
Thickness (nm)	50	500	200	150
Band-gap width E_g (eV)	1.3	0.95	3.2	3.5
Electron affinity χ (eV)	4	4.2	4.2	4
Relative permittivity ε_r	20	20	15	10
Conduction-band effective density of states N_C (cm^{-3})	2×10^{19}	5×10^{17}	1×10^{20}	1×10^{22}
Valence-band effective density of states N_V (cm^{-3})	4×10^{19}	5×10^{17}	1×10^{20}	1×10^{22}
Electron mobility μ_n ($cm^2/V{\cdot}s$)	5×10^{-2}	Tunable	5	20
Hole mobility μ_p ($cm^2/V{\cdot}s$)	5×10^{-2}	Tunable	2.5	—
Donor impurity atom density N_D (cm^{-3})	1×10^{14}	—	Tunable	1×10^{20}
Acceptor impurity atom density N_A (cm^{-3})	Tunable	1×10^{14}	—	—

Table S1 summarizes all fixed material parameters for the SCAPS simulation of the four functional layers (ITO, ZnO, PbS, PbS-EDT) of the PIN detector. All physical constants are referenced from published literature on lead sulfide quantum dot optoelectronics. Adjustable parameters such as thickness and doping are determined through scanning to achieve the optimal absorption layer thickness of 450 nm in the main text. The complete set of parameters is used to support the analysis of the difference in external quantum efficiency between simulation and experiment in Section 2.1 of the main text.

Table S2. Complete parameter list of the low-noise signal conditioning circuit.

Circuit module	Device model / Component value	Function description
TIA input op-amp	OPA847	Low-noise wideband JFET op-amp for I-V conversion
Secondary amplification & filter op-amp	ADA4625	High-speed voltage amplification
Feedback resistor R_f	40 kΩ	Transimpedance gain setting
Resistor R_1	1 kΩ	Input resistance of secondary amplifier
Resistor R_2	10 kΩ	Feedback resistance of secondary amplifier
Resistor R_3, R_4	500 Ω each	Isolation resistance for filter stage
Compensation capacitor C_1	2 pF	Eliminate high-frequency oscillation of TIA
Filter capacitors C_2, C_3	1 nF each	Second-order Butterworth low-pass filter

Table S2 records all the hardware specifications of the self-developed two-stage transimpedance amplifier circuit. The first stage utilizes the OPA847 to convert nA-level weak photocurrent into voltage; the second stage employs the ADA4625 to perform gain amplification and low-pass filtering. The feedback resistor, 40 kΩ, was selected after multiple sets of gain-bandwidth-noise trade-off tests. The total transimpedance gain of the circuit is 4×10^5 V, with a -3 dB bandwidth of 337 kHz, which matches the bandwidth of the lead sulfide detector. Direct output-noise spectral measurement of this circuit is limited by test equipment, and low-noise performance is verified via component-level selection and system-level imaging results.

Table S3. Quantitative image-quality metrics of reconstructed SWIR images under gradient incident optical power corresponding to Figure S4. The reconstructed image obtained at 0.7 mW is taken as the ground-truth reference. All metrics are calculated pixel-by-pixel from raw 128 × 128 reconstructed gray-scale matrices without manual denoising or brightness adjustment.

Incident Optical Power	SNR (dB)	PSNR (dB)	SSIM
0.1 mW (1st image)	7.01	15.68	0.422
0.3 mW (2nd image)	11.24	19.41	0.574
0.5 mW (3rd image)	16.05	23.97	0.753
0.7 mW (4th image)	Ground-truth reference	Ground-truth reference	Ground-truth reference

This table lists the quantitative evaluation results for low-light imaging tests. Only the incident SWIR optical power was varied, while optical path, DMD projection parameters, signal-conditioning circuit, and data-acquisition settings were kept unchanged. With the increase of incident optical power, SNR, PSNR, and SSIM increase monotonically, reflecting the improvement of reconstructed-image fidelity. A distinguishable target can be reconstructed even at the minimum incident power of 0.1 mW, which experimentally demonstrates the low-light imaging capability of the PbS QD single-pixel imaging system.
